# Supplementary material for: It Takes Two to Tango: Potential Prognostic Impact of Circulating TGF-Beta and PD-L1 in Pancreatic Cancer
Source: Life (Basel). 2022 Jun 26;12(7):960. doi: 10.3390/life12070960 (PMC9323895; doi:10.3390/life12070960)
Supplement: Supplementary file 1 [file life-12-00960-s001.zip › life-1737317-SI.pdf]

life

MDPI

Supplemental Figures

A

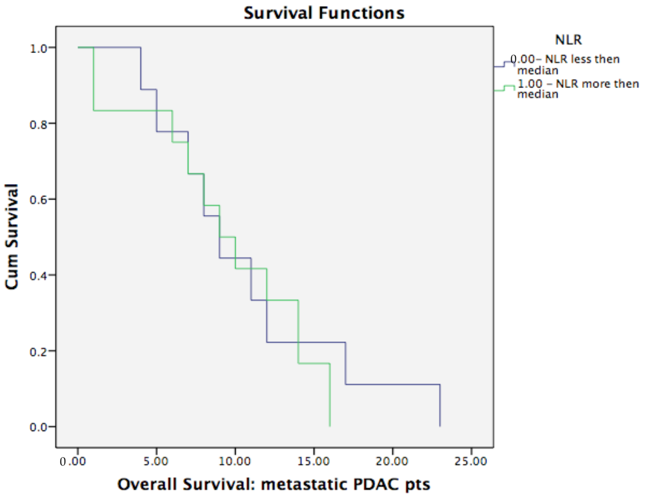

B

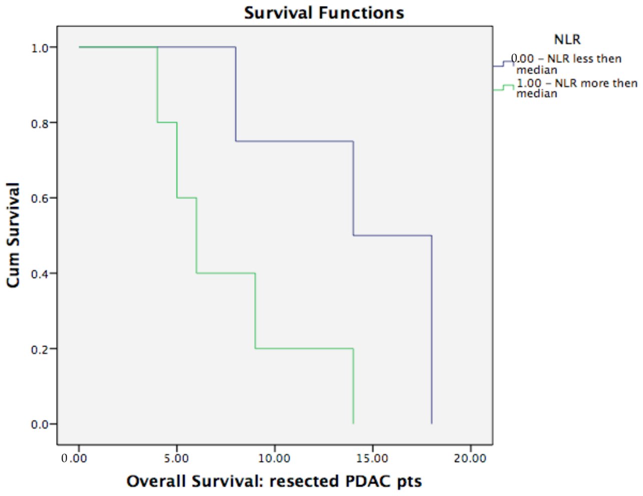

Supplemental Figure S1. OS in metastatic (A) and radically resected (B) PDAC according to values of NLR. (**A**) Kaplan–Meier curves showing no statistically significant difference for metastatic PDAC patients with upregulated (higher than median) versus downregulated (lower than median) baseline NLR values (Logrank test, $p = 0.6$). (**B**) Kaplan–Meier curves showing no statistically significant difference for radically resected PDAC with upregulated (higher than median) versus downregulated (lower than median) baseline NLR values (Logrank test, $p = 0.058$).
